# Patient and clinician perspectives of a remote monitoring program for COVID-19 and lessons for future programs

**DOI:** 10.21203/rs.3.rs-2234197/v1

**Published:** 2022-11-22

**Authors:** Krisda Chaiyachati, Judy Shea, Michaela Ward, Maria Nelson, Medha Ghosh, Julianne Reilly, Sheila Kelly, Deena Chisholm, Zoe Barbati, Jessica Hemmons, Dina Abdel-Rahman, Jeffrey Ebert, Ruiying Xiong, Christopher Snider, Kathleen Lee, Ari Friedman, Zachary Meisel, Austin Kilaru, David Asch, M. Kit Delgado, Anna Morgan

**Affiliations:** University of Pennsylvania; University of Pennsylvania; University of Pennsylvania; University of Pennsylvania; University of Pennsylvania; University of Pennsylvania; University of Pennsylvania; University of Pennsylvania; University of Pennsylvania; University of Pennsylvania; University of Pennsylvania; University of Pennsylvania; University of Pennsylvania; University of Pennsylvania; Comcast NBCUniversal; University of Pennsylvania; University of Pennsylvania; University of Pennsylvania; University of Pennsylvania; University of Pennsylvania; University of Pennsylvania

## Abstract

COVID Watch is a remote patient monitoring program implemented during the pandemic to support home dwelling patients with COVID-19. The program conferred a large survival advantage. We conducted semi-structured interviews of 85 patients and clinicians using COVID Watch to understand how to design such programs even better. Patients and clinicians found COVID Watch to be comforting and beneficial, but both groups desired more clarity about the purpose and timing of enrollment and alternatives to text-messages to adapt to patients’ preferences as these may have limited engagement and enrollment among marginalized patient populations. Because inclusiveness and equity are important elements of programmatic success, future programs will need flexible and multi-channel human-to-human communication pathways for complex clinical interactions or patients who do not desire tech-first approaches.

## Background

Patients with COVID-19 (coronavirus disease) can experience rapid and unpredictable clinical deterioration. This concern was heightened in the early months of the pandemic when the clinical course of COVID-19 was unknown, treatment was entirely supportive, and vaccines were unavailable. Simultaneously, office-based outpatient practices largely used telephone or telemedicine encounters to provide guidance for providing reassurance or managing symptoms at home or directing patients with concerning symptoms to acute care settings. To manage the large volumes of encounters, particularly during high community case counts, several health systems developed remote patient monitoring programs to support home dwelling COVID-19 infected patients.^1–9^

Our 6-hospital health system with over 500 outpatient practices enrolled adult patients with test-confirmed COVID-19 or symptoms of COVID-19 in COVID Watch, a 14-day remote patient monitoring program which resulted in lower patient mortality compared to matched control patients not enrolled in the program.^7,10^ While the program was clinically effective, we elected to ask patients and clinicians how it could be improved. Their perspectives could lead to improvements in this program or in remote engagement programs more generally.^11–15^

This study investigates the perspectives of patients and provider groups who interacted with COVID Watch: (1) patients enrolled in the program, (2) primary care and Emergency Department (ED) clinicians who enrolled patients in or had their COVID-19 patients managed by COVID Watch; and (3) administrators in primary care or the Emergency Department. The study’s aim was to understand patients’ and clinicians’ experiences interacting with the COVID Watch program, how the program could be improved, and lessons from COVID Watch that could be extended to the design and implementation of future remote patient monitoring programs.

## Methods

### COVID Watch Overview

Patients were enrolled in COVID Watch by outpatient or ED clinicians through an application embedded in Penn Medicine’s electronic health record. Patients were additionally offered COVID Watch via an automated text message if they received a positive result from a COVID-19 test conducted by a Penn Medicine laboratory.

Once enrolled, COVID Watch sent twice daily text messages in English or Spanish that asked “How are you feeling compared to 12 hours ago: better, same, or worse?” Patients who replied “worse” were subsequently asked, “Is it harder than usual for you to breathe: yes or no?”. Patients who responded “yes” generated an electronic health record (EHR) alert, monitored by a team of telemedicine clinicians (nurses, advanced practice providers, and physicians) available 24/7, who contacted the patient within one hour. Interpretation services were used when needed. Patients could also text the word “worse” at any time to connect with a clinician, also within one hour. During the study period, some participants were automatically randomized to receive a home fingertip pulse oximeter or not.^10^ Those who received a pulse oximeter were asked to report their symptoms and their ambulatory oxygen saturation levels. Patients whose oxygen saturation, SpO_2_, was below 90% or had decreased by more than 3% and below 95%, were called by the clinical staff who triaged the patient using standardized protocols. The program was free and did not require a patient to have established care with a Penn Medicine provider (e.g., primary care) or insurance. We describe the COVID Watch interventions, including the pulse oximeter trial, in previous publications.^7,10,16^

## Study Participants And Setting

We conducted semi-structured interviews with three groups of people who interacted with COVID Watch:(a) patients who had been enrolled in COVID Watch within the prior 90 days, (b) primary care and ED clinicians who directly enrolled patients in COVID Watch or had their patients enrolled into COVID Watch, and (c) administrators in primary care or the ED. Ethical approval was provided by the Institutional Review Board at the University of Pennsylvania.

## Patient Recruitment And Sampling Strategy

Patients were recruited via phone between February and June 2021, a timespan that included the randomized controlled trial of fingertip pulse oximetry among COVID Watch enrolled patients.^10^ Patients were purposively sampled across two patient-level strata to gain a diversity of patient perspectives on their COVID Watch experience: (a) having a pulse oximetry device mailed to their house (or not), and (b) level of engagement in the program (high vs. low, defined as responding to twice-daily, automated text message prompts at least 10 out of 14 days of enrollment versus fewer). The cutoffs were based on the median level of engagement. We monitored and recruited patients to attempt balance across racial, ethnic, and language sub-groups. Patients provided verbal informed consent were compensated $50 USD (United States dollar) for their time.

## Clinician And Administrator Recruitment And Sampling Strategy

Clinicians (e.g., physicians, nurse practitioners, and physician assistants) and administrators (e.g., medical directors, physician leads, or non-clinical practice managers) were recruited using emails between July and November 2021. Clinicians and administrators were purposively sampled across two health system level strata in hopes of gaining a diversity of provider perspectives of COVID Watch: (a) primarily worked in an ED setting vs primary care setting, and (b) providers that enrolled a high versus low number of patients in COVID Watch. For both settings, high enrolling clinicians were defined as those who enrolled ≥ 15 patients and low enrollers were defined as less than 10 patients. These cutoffs were based on the median level of enrollment. Administrators were recruited from the clinical sites of the providers. Snowballing techniques were used to identify additional clinicians or staff who might have been influential in encouraging clinicians or staff members to enroll patients in COVID Watch. Clinicians and administrators provided verbal informed consent and were compensated $50 USD for their time. Interviews were conducted before the publication of COVID Watch’s evaluation.^7,10^

## Interview Guide Development

We created three semi-structured, open-ended interview guides (see Table 1, **Supplementary 1, Supplementary 2, Supplementary 3**) for each cohort: patients, providers, and administrators. Guides were created by core members of the research team (KHC, JS, MW, MN, JR, MKD, AF, SK, MG, AM) and reviewed by the larger team. They were pilot tested with at least two participants in each cohort. Questions were open-ended and included follow-up probes to allow participants to expand upon answers. After the interview, participants self-reported sociodemographics.

## Data Collection And Analysis

Researchers (MW, MN, and ZB) conducted audio-recorded phone interviews in English or Spanish. Verbal informed consent was obtained prior to all interviews. English and Spanish audio recordings were transcribed by Datagain Services (Seacaucus, NJ), with the Spanish audio transcribed into English. Transcripts were then entered into NVivo 1.5 (QSR International) for coding and analysis.

Separately for patients, clinicians, and administrators, early interview transcripts were used to develop an initial codebook using a modified content analysis^17,18^ approach that relied upon the structure of the interview guide but allowed for emergent themes. The codebook was applied to all transcripts. Recruitment and emergent findings were reviewed in bi-weekly team meetings. The achieved inter-rater reliability was ĸ=0.81 across co-coded transcripts. Patient interviews lasted an average of 53 minutes (range 37–74 minutes) and clinician and administrator interviews lasted an average of 36 minutes (range 23–54 minutes).

All study protocols and instruments were deemed exempt by the University of Pennsylvania’s Institutional Review Board.

## Results

### Participant Characteristics

In total, 85 interviews were completed. Forty-seven patients were interviewed, who were on average 50 years old and mostly female, White, non-Hispanic, and English speaking (see Table 1). Because most administrators were clinicians and no major thematic differences were identified between clinicians and administrators, we combine these groups for presentation and refer to both groups as “clinicians” henceforth. The sample of 38 clinicians was primarily female, White, non-Hispanic, and physicians, and had been in practice for 11 years or more (see Table 2). Across all cohorts, themes aligned into three categories: (1) sentiments about COVID Watch, (2) feedback for improving COVID Watch and only asked of clinicians, (3) lessons learned from COVID Watch that have implications for future remote patient monitoring programs (see Table 3). There was no notable thematic difference by level of patient engagement or clinicians’ predilection (high vs. low) for enrolling patients into COVID Watch, therefore themes are aggregated across strata. Results specific to patients with pulse oximetry devices and clinicians’ practice setting (ED vs. primary care) are noted below. Quotes to illustrate each theme are presented in Table 4 for patients and Table 5 for clinicians.

## Sentiments About Covid Watch

### Patient Perspectives Comforting

Most participants described text messages as a comforting reminder that healthcare professionals were monitoring their well-being. COVID Watch was viewed as a positive alternative to being admitted and monitored in a hospital inpatient setting. Even among individuals who never required the support of a clinician, being able to contact a clinician or escalate their care while they had COVID-19 provided peace of mind. Participants also appreciated that the regular text messages helped them monitor their symptoms over time and track when their symptoms were improving.

### Irritating

Some participants felt that the text messages were excessive, intrusive, or annoying. These feelings were common for patients at the ends of the illness spectrum, either with mild to no symptoms, or conversely so ill they did not have the energy to respond to messages in a timely manner. Those who had a low response rate to the twice daily messages were often participants who felt the text messages were unhelpful.

### Insufficient

Some patients who were worried about their COVID-19 symptoms or those who were fearful about the risk of severe illness expressed a desire for real-time support from human clinicians instead of the automated, routine text messages provided by COVID Watch. Some participants wanted to report additional symptoms beyond feeling short of breath.

### Clinician Perspectives Comforting

ED clinicians often described taking comfort in knowing that COVID Watch could monitor patients discharged home. Many described it as a valuable “safety net” for patients especially when COVID-19 was a new illness. This feeling of a safety net was particularly true in clinicians’ discussions of patients with significant social needs (e.g., without a primary care provider, socially isolated) and patients who did not meet admission criteria but whom they worried might decompensate at home after being discharged from the ED.

ED and primary care clinicians believed that COVID Watch also gave their patients a sense of comfort. They knew the program would monitor them and provided an alternative to the ED as the sole source of COVID-19 care.

Some clinicians saw the provision of pulse oximetry devices to use at home as an important way to give providers and patients additional data points about the severity of a patient’s illness. For example, one ED clinician described being able to trust a patient’s report of dyspnea more if the patient used a pulse oximeter at home.

ED clinicians did not believe that the ability to enroll patients in the program influenced their decisions to admit versus discharge.

### Increased Access to Care for Patients

COVID Watch’s accessibility was seen as a key benefit to the program. Patients had quick and easy access to a clinician if needed; patients were not alone in their health decision-making; the program alleviated patient fear of the unknown; and the program was free of charge. Additionally, a few clinicians reported COVID Watch increased access for their Spanish-speaking patients, which was described as a key need at some sites.

### Reduced Follow-up Burden

Many primary care clinicians found COVID Watch to be an important tool for managing follow-up care when the volume of patients’ needs was high.

## Feedback For Improving Covid Watch

### Patient Perspectives Improve the Enrollment Process

Some patients did not recall when or how they were enrolled in COVID Watch. Patients who knew they were enrolled tended to describe more positive feelings about starting the program.

A misconception about the program was that some patients thought it was their own doctor who had enrolled them in COVID Watch and was personally monitoring their symptoms.

### Clarify the Monitoring and Escalation Process

Patients desired clearer, more concise information about what symptoms would result in clinical escalations and found the subjective nature of the daily text message (e.g., “Are your symptoms the same, better, or worse than 12 hours ago”) to be challenging. Some patients desired more quantitative measures such as a 0–10 number scale for their dyspnea. This desire for quantitative measures was also reflected in patients’ positive reception to the pulse oximetry device. The device provided an objective measure that enabled patients to feel more confident about their clinical course.

Some patients expressed a preference for phone calls over text messages, referencing the difficulty that older patients can have with texting, or not having phones that are equipped for text messaging. Other patients thought phone calls would be preferable because they would give clinicians more clarity about how patients are feeling.

Spanish-speaking patients more often felt COVID Watch was not able to fully meet their needs. Some Spanish speakers expressed how Spanish-speaking cultures tended to be more phone-call oriented, and so an option to choose the modality of the messages may provide a better cultural fit for some.

### Clinician Perspectives Improve the Enrollment Process

Clinicians tended to describe the process of enrolling into COVID Watch as relatively easy, but there was a desire to make enrollment even easier. Some believed it was tedious to, for example, go into the patient’s exam room to ensure patients received the program’s initial text message or to ensure the patient’s phone number was correct. In addition, some clinicians felt that their own familiarity with the enrollment process waned if they had not enrolled a patient recently. Some also expressed uncertainty about the program’s details such as the ability to enroll patients over the weekends.

### Provide Solutions for Patients with Limited Device Access or Hesitancy

Clinicians highlighted barriers related to patients’ accessing COVID Watch and hesitancy to use their phones for engaging in care. Participation required the ability to use a cell phone with text messaging. Access to the required technology was particularly challenging for elderly patients (especially those who lived alone) and patients experiencing homelessness.

### Address Low-Literacy and Language Preferences Among Patients

A key access-related barrier clinicians discussed was that the program required comfort in reading and writing in English or Spanish; other languages should be considered. Other enhancements for accessibility included offering an option for patients to use a landline; offering access to a central hotline phone number those patients could call; or distributing cell phones for patients to use. Finally, some clinicians recommended that patients be given the ability to enroll themselves.

### Create a Feedback Loop for Clinicians

ED and primary care clinicians discussed a desire to know the clinical course of their patients after enrollment in COVID Watch and were interested in knowing which patients did not escalate, those who escalated to a COVID Watch nurse, or those who unenrolled from the program early. This would serve as a mechanism to inform clinicians about the quality of their care, particularly if enrolling in COVID Watch was effective, and a reminder that the COVID Watch program was still enrolling patients.

## Lessons For Future Of Remote Patient Monitoring Programs From Clinicians

### Enhanced Data Collection

Clinicians felt that remote patient monitoring programs will be an important part of practicing medicine in the future. However, data collected should have concrete benchmarks. Clinicians were hesitant about COVID Watch’s subjective self-reports and felt more objective measures should be used in future programs, COVID-19 related or otherwise.

Both ED and primary care clinicians felt remote patient monitoring programs should provide patients with the appropriate health data collection tools, like a home pulse oximeter or blood pressure cuff, to collect and report data back to their healthcare team. Some also suggested greater integration with existing electronic health records, directly embedding remotely recorded results into the medical record.

### A Guide for Patients

ED clinicians felt that remote patient monitoring could reduce the number of ED visits by giving patients more accurate, objective data about when not to come to the ED. By using objective data and clear cutoffs, patients could be clearly guided to seek the right level of care. In addition, the ability to provide reassurance to patients with a remote monitoring program might help patients being discharged home from the ED.

### Extend Remote Patient Monitoring to Non-COVID-19 Conditions

Many clinicians also felt remote patient monitoring will be particularly valuable for certain chronic and acute conditions. For example, participants tended to perceive targeted data collection to be practical and effective for monitoring and treating conditions like congestive heart failure, diabetes, asthma, weight management, and post-surgery recovery. To evolve for other use cases, however, clinicians emphasized that remote patient monitoring tools needed to be easy to use and equitable for patients, and clinicians must be confident in the quality of the data collected.

## Discussion

Overall, while patients and clinicians found COVID Watch to be comforting and beneficial, improvements to the design and implementation of the program will be important for the program’s future and have implications for the design of future remote patient monitoring programs.

COVID Watch often provided a sense of comfort and reassurance to both patients and clinicians, facilitated by increased access to care because of its free cost and Spanish-language availability. Despite their enthusiasm, patients and clinicians desired a better user experience. Patients wanted more clarity about when and how they were enrolled in the program. The confusion over enrollment may have contributed to lower engagement in the program, and also may have lowered perceptions of its utility. Patients and clinicians also desired the program to build in flexibility, expressing concerns about the universal use of automated text messages for patients. For example, the program may have had limited uptake among patients who had reticence about using technology to communicate their health needs, limited English or Spanish literacy, or preferred additional languages. These subgroups are among those who are at increased risk for limited access to care and experienced worse COVID-19 outcomes.^19^

Our findings have generated three key insights for future remote patient monitoring programs to manage COVID-19 or other clinical conditions. First, remote patient monitoring programs should not be static, one-time builds or implementations. While these programs may have automation or use digitized algorithms, they are human facing programs that should evolve as technology advances, patient and clinician expectations of technology evolve, and standards for managing targeted disease conditions change. Developing systems for monitoring program performance and patient engagement and seeking patient and clinician feedback to continuously re ne these programs are as important as achieving intended health outcomes.

Second, health systems must acknowledge the human resources needed to support remote patient monitoring programs, even if automation is embedded in the program. While automation can improve efficiency for some patients, successful programs will need to marry technology with options for human interaction. Tech-first approaches may not always be welcomed. Some patients in our study indicated the desire to connect with a human clinician, wanting to avoid the automated text message system more generally. Yet, at the same time, automation allowed for a team of 3–4 nurses to simultaneously manage efficiently and effectively over one thousand patients during the staffing shortages of the pandemic,^7^ a challenge projected to remain over the coming years.^20,21^ Using default pathways (e.g., text messages) that are automated, complemented by alternative pathways (e.g., interactive voice recordings or human-to-human phone calls) that are customized to the user’s needs, may be one solution for greater engagement while not overburdening current clinicians.

Finally, future programs must be designed with equity as a primary principle, recognizing patients who have the most limited access to care may need additional design considerations. For example, programs should be offered in multiple languages. In our study both patients and clinicians expressed concerns that vulnerable populations may have been excluded because more direct human-to-human connections (e.g., telephone calls) were not made available. Developing programs that correctly balance patients’ desires for human-to-human connections, promoting inclusivity, with the efficiency gains of automated processes will be important for future remote patient monitoring programs.

This study has limitations. Our analysis took place in one large academic institution. However, the institution includes six hospitals and over 500 outpatient practices across a wide geographic area, allowing us to sample participants from multiple hospitals, encompassing urban and suburban settings across the large catchment area. These interviews took place relatively early in the pandemic – within the first year – and therefore reflect the stress that both patients and clinicians felt when faced with an unprecedented crisis. Finally, patients’ and clinicians’ experiences with COVID Watch and COVID evolved during the study period such as the implementation of automated and opt-out enrollment in the fall of 2020. In addition, surges of infection, the increased availability of vaccination, and effective treatments might have influenced our participants’ responses.

Remote patient monitoring programs are a growing form of health care delivery and are being tested in a variety of clinical conditions including hypertension management, in-home administration of chemotherapy, and transitions between hospital and home. Success of these programs hinges on user-centered design to enhance experiences for both patients and clinicians^22^ as well as intentional design for traditionally marginalized groups who have not historically been considered as early adopters of new technology-based care programs.^23–25^ Understanding how a diverse group of patients engage with and experience remote patient monitoring programs, and how providers integrate them into daily workflow and clinical decision-making will be informative for future remote patient monitoring programs.

## Figures and Tables

**Table 1:** Patient and Clinician Interview Question Examples According to Themes in Participant Responses

	Examples: Patient Questions	Examples: Clinician Questions
**Sentiments**	Tell me how you felt when you got the text messages each time from the program. *Prompt*. What made you feel that way? What role did COVID Watch play in helping you manage your symptoms, if any?	What led you or your clinical team to use or not use COVID Watch? What was it like to have a patient in COVID Watch?
**Feedback**	What kinds of changes do you think the program needs to make to be more useful for patients in the future?	What recommendations do you have for improving COVID Watch? *Prompt*. What would have made it more useful to you? *Prompt*. Tell me about any frustrations or difficulties with any aspect of it. *Prompt*. What other thoughts do you have about the process, for example about things like the amount of time it took, or the ease of enrolling?
**Lessons for Future Remote Patient Monitoring Programs**	*This line of questioning was not asked of patient participants*	Can you share any “lessons learned” you have had from your experience with COVID Watch that might be relevant for future remote patient monitoring programs? How do you think remote patient monitoring programs could influence your clinical practice in the future? Are there particular areas or conditions that you think remote patient monitoring is most useful for? What parts of COVID Watch’s remote patient monitoring program do you think was the most useful for patients? For you? What aspects of COVID Watch do you think will be important for future remote patient monitoring programs?

**Table 2:** Patient Characteristics

Characteristics	N = 47
Age, mean (SD)	50 (15)
Gender, no. (%)	
Female	32 (68)
Male	15 (32)
Race, no. (%)	
White	19 (40)
Black	16 (34)
Other	12 (26)
Ethnicity, no. (%)	
Hispanic or Latino	6 (13)
Non-Hispanic or Latino	41 (87)
**Preferred language, no. (%)**	
English	40 (85)
Spanish	7 (15)
**Enrollment location, no. (%)**	
Emergency Department	21 (45)
Outpatient Setting	26 (55)
**Access to a Pulse Oximeter, no. (%)**	
Yes	34 (72)
No	13 (28)

**Table 3:** Clinician and Administrator Characteristics

Characteristics	Total	ED Clinicians	Primary Care Clinicians	ED Administrators	Primary Care Administrators
No. of participants	38	9	16	9	4
Gender, no (%)					
Female	22 (58)	2 (22)	13 (81)	4 (44)	3 (75)
Male	16 (42)	7 (78)	3 (19)	5 (56)	1 (25)
Race, no (%)					
White	33 (87)	8 (89)	14 (88)	9 (100)	2 (50)
Asian	4 (11)	1 (11)	1 (6)	0 (0)	2 (50)
Other	1 (3)	0 (0)	1 (6)	0 (0)	0 (0)
Ethnicity, no (%)					
Hispanic Latino	0 (0)	0 (0)	0 (0)	0 (0)	0 (0)
Non-Hispanic Latino	38 (100)	9 (100)	16 (100)	9 (100)	4 (100)
Provider Type, no (%)					
Physician	22 (58)	8 (89)	7 (44)	4 (44)	3 (75)
Physician Assistant	3 (8)	1 (11)	2 (13)	0 (0)	0 (0)
Nurse Practitioner	5 (13)	0 (0)	7 (44)	0 (0)	1 (25)
Years in Clinical Practice, no (%)					
< 5 years	4 (11)	1 (11)	3 (19)	0 (0)	0 (0)
5–10 years	13 (34)	1 (11)	7 (44)	4 (44)	0 (0)
11 −20 years	8 (21)	4 (44)	1 (6)	2 (22)	2 (50)
>20 years	13 (34)	3 (33)	5 (31)	3 (33)	2 (50)

**Table 4:** Summary of Patient Themes and Illustrative Interview Excerpts

	Illustrative Patient Excerpts
**Sentiments about Covid Watch**	
Comforting	“[I stayed enrolled because] it was nice knowing that there was a medical professional out there who was aware of my situation and I still, I knew if anything went wrong that, I would be able to be quickly assessed and figure out what next steps would be.” (*English speaking, White/Non-Hispanic participant*)
Irritating	“Sometimes they’d text me when I was really tired, but I think I text the wrong response saying I couldn’t breathe or something like that. I don’t know. I was half asleep when I responded, so I think the text is okay. I think it’s cool, but I think they should make phone calls instead of texting … I just thought they was annoying, but I still responded.” (*English speaking, Black/Non-Hispanic participant*)
Insufficient	“I just think the phone call could’ve been better because they also would [hear] how you sound as well, because sometimes people can’t really – they hear how they sound. I think that helps as well.” (*English speaking, Black/Non-Hispanic participant*)
**Feedback for Improving COVID Watch**	
Improve the Enrollment Process	“I wish everybody was able to get it. I don’t understand why [Family Member Name] was the only one who received it and we’re all Hospital System patients] … I think they should or at least have an option for [a patient to say no … I just say for the future, if this continues the way it does, it’s a great feature for folks who are homebound and can’t see a doctor.” (*English speaking, White/Non-Hispanic participant*)
Clarify the Monitoring and Escalation Process	“When it says like, ‘if you’re feeling worse, go to the emergency room.’ Well, what does that mean? Like, what level is worse?” (*English speaking, White/Non-Hispanic participant*)

**Table 5:** Summary of Clinician Themes and Illustrative Interview Excerpts

	Illustrative Clinician Excerpts
**Sentiments about Covid Watch**	
Comforting	“Knowing that the patients are going to have guaranteed follow-up is a huge - it makes it much more comfortable discharging those borderline patients and knowing that that follow-up will be daily and continuous.” (*Emergency department administrator*)
Increased Access to Care for Patients	“I used it a lot, because it gives me something for patients who I don’t have a relationship, it gives me some way to hand them off as a safety net for their care. A lot of my patients don’t have primary care, don’t have access to the system I don’t follow them longitudinally. So, it really was a nice mechanism for them to be at least tied in for care during their very nervous time when they had COVID.” (*Emergency department clinician*)
Reduced Follow-up Burden	“[COVID Watch] really let me focus on other patients and not following up with the same [ones]. I know that sounds tough, but timing is always difficult. And we always have more patients call in, so it kind of allowed me to pass off the COVID-positive patient, knowing that someone was going to check on them no matter what.” (*Primary care clinician*)
**Feedback for Improving COVID Watch**	
Improve the Enrollment Process	“I think [enrolling patients] was like a little bit of a struggle in the beginning. It can be hard to find the right part of the EMR where you enroll patient with the COVID Watch… [when] I started trying to use it on my own, little bit of a struggle, but then I got like another email from [Colleague] and it solidified how to use it. And then I started using it more regularly. Having said that, even now, I still sometimes can’t find how to enroll patients and so I have to like, look for it a little bit, but it only takes me a few clicks before I find it.” (*Primary care clinician*)
Provide Solutions for Patients with Limited Device Access or Hesitancy	““Maybe make it if the patient doesn’t know how to text, you guys have someone call or don’t make them text… it might be beneficial to have like, it’s like an 85-year-old that still living by themselves can’t text, they switch it to a phone call.” (*Primary care clinician*)
Address Low-Literacy and Language Preferences Among Patients	“Having access to other languages would have been really meaningful. And I think that there was definitely some wide loss in not having other languages available…” (*Emergency department clinician*)
Create a Feedback Loop for Clinicians	“[I’d suggest] a report at the end. I don’t know if you’d want to do it every day, but maybe once a week, or once every two weeks, [send] a report of what patients were reached out to and if you have any issues or improving, just so that we’re aware that it is still being done.” (*Primary care clinician*)
**Clinician Perspectives on the Future of Remote Patient Monitoring**	
Enhanced Data Collection	“I thought that the implementation of the home pulse oximeter was really helpful. Because I felt like people would say that they were short of breath, but then their number was reassuring. We expected people to get short of breath and we expected people to have some discomfort, but having a very clear number that they could use was helpful. I think that that’s actual data that’s being referred back to the nurse and/or chat system, you know… That enabled the other side of the message to get real information rather than ‘I feel’, and…[being able to] give objective data is obviously helpful.” (*Emergency department clinician*)
A Guide for Patients	“Knowing when [a patient] needs to get escalated to a phone call is important. In other words, when [patient care] needs to move off the texting medium and move away from a text bot and towards just a conversation on the phone. Having the right threshold there is important. [With COVID Watch]…there were…even more robust contact with healthcare providers.” (*Emergency department administrator*)
Extend Remote Patient Monitoring to Non-COVID-19 Conditions	“From an Emergency Department aspect, I mean, the one I guess– from other types of infections, so not just COVID, but anyone we discharge on antibiotics, we could do kind of a sepsis initiative kind of thing to prevent progression of illness and to prevent antibiotic failure, so kind of check in…So I would say, off the top of my head, that’s probably the highest yield from an Emergency Department perspective and can probably prevent readmissions and even prevent death, potentially.” (*Emergency department leader*)

## Data Availability

The datasets generated and/or analyzed during the current study are not publicly available due to the researchers not compiling or analyzing any datasets as part of this qualitative research, but additional information from the corresponding author is on reasonable request.

## Abbreviations

COVID, COVID-19: Coronavirus disease, SARS-CoV-2 (virus)
ED: Emergency Department
EHR: electronic health record
SpO_2_: oxygen saturation
USD: ($) United States dollar

## References

[1] Pronovost PJ, Cole MD, Hughes RM. Remote Patient Monitoring During COVID-19: An Unexpected Patient Safety Benefit. Jama. 2022;327(12):1125–1126. 35212725 10.1001/jama.2022.2040

[2] Aalam AA, Hood C, Donelan C, Rutenberg A, Kane EM, Sikka N. Remote patient monitoring for ED discharges in the COVID-19 pandemic. Emerg Med J. 2021;38(3):229–231. 33472870 10.1136/emermed-2020-210022

[3] Grutters LA, Majoor KI, Mattern ESK, Hardeman JA, van Swol CFP, Vorselaars ADM. Home telemonitoring makes early hospital discharge of COVID-19 patients possible. J Am Med Inform Assoc. 2020;27(11):1825–1827. 32667985 10.1093/jamia/ocaa168 PMC7454667

[4] Bell LC, Norris-Grey C, Luintel A, Implementation and evaluation of a COVID-19 rapid follow-up service for patients discharged from the emergency department. Clin Med (Lond). 2021;21(1):e57–e62. 33355255 10.7861/clinmed.2020-0816 PMC7850184

[5] Patel H, Hassell A, Cyriacks B, Fisher B, Tonelli W, Davis C. Building a Real-Time Remote Patient Monitoring Patient Safety Program for COVID-19 Patients. Am J Med Qual. 2022. 10.1097/JMQ.0000000000000046 PMC9241558 35213860

[6] Tabacof L, Kellner C, Breyman E, Remote Patient Monitoring for Home Management of Coronavirus Disease 2019 in New York: A Cross-Sectional Observational Study. Telemed J E Health. 2021;27(6):641–648. 33232204 10.1089/tmj.2020.0339

[7] Delgado MK, Morgan AU, Asch DA, Comparative Effectiveness of an Automated Text Messaging Service for Monitoring COVID-19 at Home. Ann Intern Med. 2022;175(2):179–190. 34781715 10.7326/M21-2019 PMC8722738

[8] Greenhalgh T, Knight M, Inda-Kim M, Fulop NJ, Leach J, Vindrola-Padros C. Remote management of covid-19 using home pulse oximetry and virtual ward support. BMJ. 2021;372:n677. 33766809 10.1136/bmj.n677

[9] Huynh DN, Millan A, Quijada E, John D, Khan S, Funahashi T. Description and Early Results of the Kaiser Permanente Southern California COVID-19 Home Monitoring Program. Perm J. 2021;25. 10.7812/TPP/20.281 PMC8784054 35348067

[10] Lee KC, Morgan AU, Chaiyachati KH, Pulse Oximetry for Monitoring Patients with Covid-19 at Home - A Pragmatic, Randomized Trial. N Engl J Med 2022. 10.1056/NEJMc2201541 PMC9006781 35385625

[11] Field MJ, Grigsby J. Telemedicine and remote patient monitoring. JAMA. 2002;288(4):423–425. 12132953 10.1001/jama.288.4.423

[12] Daly B, Nicholas K, Flynn J, Analysis of a Remote Monitoring Program for Symptoms Among Adults With Cancer Receiving Antineoplastic Therapy. JAMA Netw Open. 2022;5(3):e221078. 35244701 10.1001/jamanetworkopen.2022.1078 PMC8897754

[13] Fisher NDL, Fera LE, Dunning JR, Development of an entirely remote, non-physician led hypertension management program. Clin Cardiol. 2019;42(2):285–291. 30582181 10.1002/clc.23141 PMC6712321

[14] Salehi S, Olyaeemanesh A, Mobinizadeh M, Nasli-Esfahani E, Riazi H. Assessment of remote patient monitoring (RPM) systems for patients with type 2 diabetes: a systematic review and meta-analysis. J Diabetes Metab Disord. 2020;19(1):115–127. 32550161 10.1007/s40200-019-00482-3 PMC7270436

[15] Ong MK, Romano PS, Edgington S, Effectiveness of Remote Patient Monitoring After Discharge of Hospitalized Patients With Heart Failure: The Better Effectiveness After Transition -- Heart Failure (BEAT-HF) Randomized Clinical Trial. JAMA Intern Med. 2016;176(3):310–318. 26857383 10.1001/jamainternmed.2015.7712 PMC4827701

[16] Morgan AU, Balachandran M, Do D, Remote monitoring of patients with Covid-19: design, implementation, and outcomes of the first 3,000 patients in COVID Watch. NEJM Catalyst Innovation in Care Delivery. 2020.

[17] Hays DG, Singh AA. Qualitative inquiry in clinical and educational settings. New York, NY, US: The Guilford Press; 2012.

[18] Flick U. An introduction to qualitative research. 4th ed. Los Angeles;: Sage Publications; 2009.

[19] Macias Gil R, Marcelin JR, Zuniga-Blanco B, Marquez C, Mathew T, Piggott DA. COVID-19 Pandemic: Disparate Health Impact on the Hispanic/Latinx Population in the United States. J Infect Dis. 2020;222(10):1592–1595. 32729903 10.1093/infdis/jiaa474 PMC7454709

[20] Galvin G. Nearly 1 in 5 Health Care Workers Have Quit Their Jobs During the Pandemic. 2021; https://morningconsult.com/2021/10/04/health-care-workers-series-part-2-workforce/. Accessed May 26, 2022.

[21] Ollove M. Health Worker Shortage Forces States to Scramble. 2022; https://www.pewtrusts.org/en/research-and-analysis/blogs/stateline/2022/03/25/health-worker-shortage-forces-states-to-scramble. Accessed May 26, 2022.

[22] Nguyen MT, Garcia F, Juarez J, Satisfaction can co-exist with hesitation: qualitative analysis of acceptability of telemedicine among multi-lingual patients in a safety-net healthcare system during the COVID-19 pandemic. BMC Health Serv Res. 2022;22(1):195. 35164746 10.1186/s12913-022-07547-9 PMC8842908

[23] Culyer AJ, Bombard Y. An equity framework for health technology assessments. Med Decis Making. 2012;32(3):428–441. 22065143 10.1177/0272989X11426484

[24] Stiles-Shields C, Cummings C, Montague E, Plevinsky JM, Psihogios AM, Williams KDA. A Call to Action: Using and Extending Human-Centered Design Methodologies to Improve Mental and Behavioral Health Equity. Front Digit Health. 2022;4:848052. 35547091 10.3389/fdgth.2022.848052 PMC9081673

[25] Sieck CJ, Sheon A, Ancker JS, Castek J, Callahan B, Siefer A. Digital inclusion as a social determinant of health. NPJ Digit Med. 2021;4(1):52. 33731887 10.1038/s41746-021-00413-8 PMC7969595

